# USP45 acts as an oncogene to regulate the proliferation of esophageal cancer cells

**DOI:** 10.1016/j.gendis.2023.101135

**Published:** 2023-09-30

**Authors:** Kai Li, Qian Wang, Hua Bian

**Affiliations:** a Zhang Zhongjing College of Chinese Medicine, Henan Key Laboratory of Zhang Zhongjing Formulae and Herbs for Immunoregulation, Nanyang Institute of Technology, Nanyang, Henan 473004, China; b College of Orthopaedics and Traumatology, Henan University of Chinese Medicine, Zhengzhou, Henan 450046, China

Deubiquitinases (DUBs) are a class of enzymes that are able to reverse the process of ubiquitination.^1^ Extensive research has revealed that there are more than 100 DUBs found in humans, which are classified into seven main families: ubiquitin-specific proteases (USPs), ubiquitin carboxyl-terminal hydrolases, the Machado-Joseph disease domain superfamily, the otubain/ovarian tumor-domain containing proteins, the ZUFSP (Zinc finger with UFM1-specific peptidase domain protein) family, the JAB1/MPN/MOV34 proteases, and the novel motif interacting with ubiquitin-containing DUB family. All DUBs are cysteine proteases, except the JAB1/MPN/MOV34 proteases, which are Zn^2+^ metalloproteases.^1^ The family with the largest number is the USPs. These enzymes remove the ubiquitin molecule from the larger protein, which leads to protein stability and protection from proteasome degradation. Many USPs have been demonstrated to be closely linked with the proliferation and metastasis of tumor cells, as well as facilitating tumor immune evasion.^1^ More importantly, inhibitors targeting USPs have become a hot topic in tumor therapy, showing promising prospects.^1^

USP45 belongs to the USPs family, which contains a zinc-finger ubiquitin-specific protease domain, playing a crucial part in regulating their activity. Unlike other USPs, the role of USP45 in tumors is still largely unknown. In our previous study, a pan-cancer analysis revealed that USP45 was up-regulated in most tumor types and was negatively correlated with both overall survival and recurrence-free survival of patients.^2^ These findings suggest that USP45 acts as an oncogene. Esophageal cancer is the seventh most diagnosed cancer and the sixth most common cause of cancer-related death worldwide. Our previous study suggests that USP45 is up-regulated in esophageal cancer tissues and is closely associated with the progress of esophageal cancer.^2^ In this study, to further explore the possibility of USP45 as a therapeutic target for tumors, we detected the effect of USP45 on the proliferation of esophageal cancer cells through *in vitro* and *in vivo* studies. In esophageal cancer cells KYSE140 and KYSE410, we constructed stable cell lines of USP45 overexpression and knockdown, respectively (Fig. 1A). Based on the results of cell clone formation assays and CCK-8 assays, overexpression of USP45 significantly promoted the proliferation of KYSE140 and KYSE410 cells (Fig. 1B, C), whereas knockdown of USP45 inhibited cell proliferation (Fig. 1D, E). Furthermore, the effect of USP45 on cell proliferation was also confirmed by Western blot, which showed that overexpression of USP45 up-regulated the expression of cyclin D1, Ki-67, and PCNA (Fig. 1F), while knockdown of USP45 down-regulated cyclin D1, Ki-67, and PCNA (Fig. 1G). The *in vivo* study results are consistent with *in vitro* study results (Fig. 1H). USP45 knockdown significantly inhibited the growth rate and weight of tumors and the protein expression of cyclin D1, Ki-67, and PCNA in nude mice (Fig. 1I). Our results suggest that USP45 may be a potential target of esophageal cancer.

**Figure 1:**
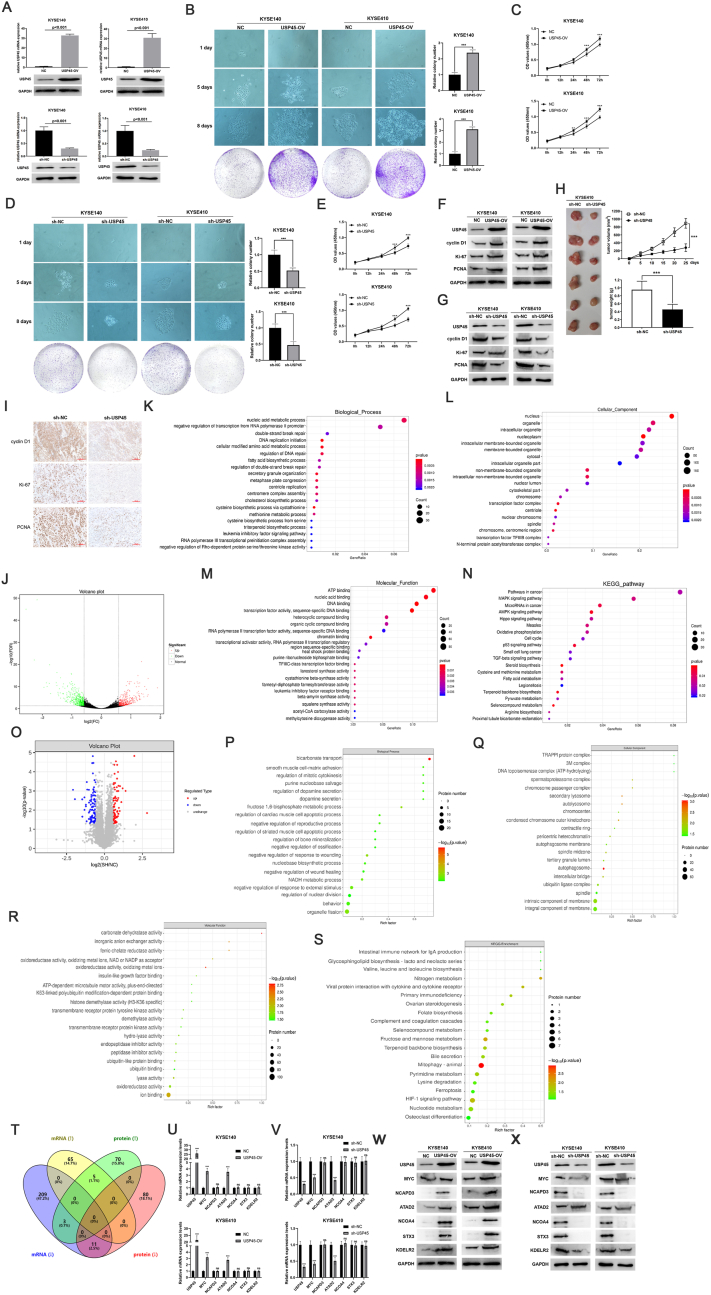
USP45 regulated the proliferation of esophageal cancer cells. **(A)** KYSE140 and KYSE410 stable cell lines of USP45 overexpression and knockdown were constructed and identified by RT-qPCR and Western blot. USP45-OV represented the overexpression of USP45, and sh-USP45 represented the knockdown of USP45. The effects of USP45 overexpression and knockdown on the proliferation of KYSE140 and KYSE410 cells were detected by cell clone formation assay **(B, D)** and CCK-8 assay **(C, E)**. The effects of USP45 overexpression and knockdown on cell proliferation-associated biomarkers cyclin D1, Ki-67, and PCNA were detected by Western blot **(F, G)**. USP45 knockdown significantly inhibited the growth rate and weight of tumors derived from (shUSP45) or (sh-NC) KYSE410 cells in nude mice **(H)** and down-regulated the protein levels of cyclin D1, Ki-67, and PCNA which were detected by immunochemistry **(I)**. **(J)** Volcano plot of transcriptome analysis based on KYSE410 stable cell lines of USP45 knockdown. Based on transcriptome sequencing, GO analysis, including biological process **(K)**, cellular component **(L)**, molecular function **(M)**, and KEGG pathway analysis **(N)** was used to perform the function enrichment of differentially expressed genes. **(O)** Volcano plot of proteomic analysis based on KYSE410 stable cell lines of USP45 knockdown. Based on proteomic, GO analysis, including biological process **(P)**, cellular component **(Q)**, molecular function **(R)**, and KEGG pathway analysis **(S)** was used to perform the function enrichment of differentially expressed proteins. **(T)** The association analysis of transcriptome and proteome was performed using the Wayne analysis tool. The effects of USP45 overexpression and knockdown on the mRNA **(U, V)** and protein **(W, X)** expression levels of MYC, NCAPD3, ATAD2, NCOA4, STX3, and KDELR2 were detected by RT-qPCR and Western blot.

As a deubiquitinating enzyme, the role of USP45 in tumorigenesis has been poorly understood. However, in our previous study, we conducted a comprehensive bioinformatics analysis and revealed that USP45 is closely associated with mRNA methylation, tumor heterogeneity, tumor stemness, and tumor immunity.^2^ However, definitive evidence for the involvement of USP45 in the development of esophageal carcinoma is lacking. In this study, we carried out transcriptome sequencing using KYSE140 stable cell lines of USP45 knockdown. Using fold change ≥1.5 and false discovery rate <0.05 as screening criteria, we have identified a total of 960 differentially expressed genes, of which 401 were up-regulated while 559 were down-regulated (Fig. 1J and Table S1). To analyze the mechanism of USP45 in esophageal cancer, we performed GO analysis and KEGG pathway analysis based on these differentially expressed genes. According to the results of the GO analysis, the functions of the differentially expressed genes were mainly related to nucleic acid metabolic processes, negative regulation of transcription from RNA polymerase II promoter, and double-strand break repair. In addition, the cellular components in which these genes were enriched were mainly localized in the nucleus (Fig. 1K–M and Table S2–4). A recent study has shown that USP45 was up-regulated in cervical cancer, promoting cervical cancer progression, cancer cell stemness, and drug resistance by regulating the deubiquitination of MYC.^3^ It is well known that MYC is a classical transcription factor, which is closely related to cell proliferation, apoptosis, and other biological processes. In this study, the results of transcriptome analysis showed that the mRNA expression level of MYC was down-regulated after knocking down USP45. Thus, USP45 may regulate MYC expression at both transcriptional and post-translational levels. In addition, our results strongly suggest that USP45 is associated with DNA repair, which is also consistent with the currently known evidence. It was shown that USP45 could inhibit the ubiquitin-proteasome degradation pathway of ERCC1 protein, a DNA repair-related nucleic acid endonuclease, thereby enhancing the ability of tumor cells to repair DNA damage.^4^ KEGG pathway analysis showed that USP45 may be associated with the MAPK signaling pathway, AMPK signaling pathway, Hippo signaling pathway, cell cycle, p53 signaling pathway, and TGF-β signaling pathway (Fig. 1N and Table S5). Currently, no studies have confirmed the correlation between USP45 and these signaling pathways, which provides a direction for future research.

Since USP45 is a member of the deubiquitinating enzyme family, it mainly regulates the deubiquitination of substrates and affects the protein stability. Therefore, we carried out proteomic analysis using KYSE140 stable cell lines of USP45 knockdown. By applying the screening criteria of fold change ≥1.5 and false discovery rate <0.05, we have identified 169 differentially expressed proteins, of which 78 were up-regulated while 91 were down-regulated (Fig. 1O and Table S6). We performed GO analysis and KEGG pathway analysis based on these differentially expressed proteins. GO analysis showed that the functions of USP45 may be related to bicarbonate transport, cell-matrix adhesion, negative regulation of wound healing, regulation of mitotic cytokines, regulation of nuclear division, and organelle fission (Fig. 1P–R and Table S7–9). These results suggest that USP45 is closely associated with cell proliferation and migration. It has been shown that USP45 is involved in cell migration by regulating the deubiquitination of SPDL1.^5^ The present study has demonstrated that USP45 can regulate cell proliferation. In addition, we found that the functions of differentially expressed proteins were mainly enriched in the autolysosome, secondary lysosome, ubiquitin ligase complex, TRAPPI protein complex, autophagosome membrane, and autophagosome. These results suggest that USP45 may be closely associated with cell autophagy. The results of KEGG pathway analysis also showed a significant correlation between USP45 and mitophagy. In addition, USP45 is also associated with the intestinal immune network, viral protein interaction with cytokine and cytokine receptor, primary immunodeficiency, ferroptosis, and HIF-1 signaling pathway, suggesting that USP45 may play an important role in biological processes such as immune response, hypoxia, and ferroptosis (Fig. 1S and Table S10). To explore the potential substrates of USP45, we performed an association analysis of transcriptome and proteomic results. Compared with the control group, there were 80 genes in the USP45 knockdown group, whose mRNA expression levels were not significantly different, but protein expression levels were down-regulated (Fig. 1T and Table S11). We speculate that these proteins may be the potential substrates of USP45. In addition, there were 11 genes whose mRNA and protein expression levels were both down-regulated (Fig. 1T and Table S11). We speculate that the 11 proteins may also be the potential substrates of USP45. Since USP45 may regulate the expression levels of substrates at both the transcriptional and post-translational levels. It has been confirmed that SPDL1 is a substrate of USP45,^5^ which is consistent with our findings. In addition, our study also showed that USP45 can regulate the mRNA expression level of SPDL1. Furthermore, through RT-qPCR and Western blot, we confirm that USP45 overexpression up-regulated the mRNA and protein levels of MYC and ATAD2, while USP45 knockdown down-regulated MYC and ATAD2. In addition, USP45 overexpression up-regulated protein levels of NCAPD3, NCOA4, STX3, and KDELR2, while USP45 knockdown down-regulated them. However, USP45 overexpression or knockdown did not affect the mRNA levels of NCAPD3, NCOA4, STX3, and KDELR2 (Fig. 1U–X). These results were consistent with transcriptomic sequencing and proteomics.

In conclusion, our study revealed that USP45 may be a new target for esophageal cancer treatment through *in vitro* and *in vivo* experiments. Through transcriptome and proteomic analyses, we preliminarily explored the mechanisms of USP45 in esophageal cancer, including cell proliferation, cell migration, immunity, ferroptosis, and autophagy. Deubiquitinating enzymes perform different functions mainly by regulating the ubiquitination of substrates, so identifying substrates of USP45 is a key component of future research. In the present study, through transcriptome and proteomic association analysis, we identified 91 proteins as possible substrates of USP45. However, further studies are required to validate the mechanism of USP45 in esophageal cancer due to the sensitivity of proteomic analysis and the limitations of specific cell lines.

## Ethics declaration

The study protocol is approved by the Ethics Committee of Nanyang Institute of Technology (the ethics approval code: NYISTIRB2021-003).

## Author contributions

Kai Li and Hua Bian designed the study. Kai Li, Qian Wang, and Hua Bian performed the experiments and data analysis. Kai Li and Hua Bian wrote the manuscript. All authors contributed to the article and approved the submitted version.

## Data availability

All the results are presented in the article and Supplementary Material. Further inquiries can be directed to the corresponding authors.

## Conflict of interests

The authors declare no potential conflict of interests.

## Appendix A Supplementary data

Multimedia component 1 Multimedia component 1

Table S1 The list of 960 differentially expressed genes in transcriptome analysis. Table S1

Table S2 The full list of GO enrichment analysis (biological process) based on 960 differentially expressed genes. Table S2

Table S3 The full list of GO enrichment analysis (cellular component) based on 960 differentially expressed genes. Table S3

Table S4 The full list of GO enrichment analysis (molecular function) based on 960 differentially expressed genes. Table S4

Table S5 The full list of KEGG pathway analysis based on 960 differentially expressed genes. Table S5

Table S6 The list of 169 differentially expressed proteins in proteomic analysis. Table S6

Table S7 The full list of GO enrichment analysis (biological process) based on 169 differentially expressed proteins. Table S7

Table S8 The full list of GO enrichment analysis (cellular component) based on 169 differentially expressed proteins. Table S8

Table S9 The full list of GO enrichment analysis (molecular function) based on 169 differentially expressed proteins. Table S9

Table S10 The full list of KEGG pathway analyses based on 169 differentially expressed proteins. Table S10

Table S11 The list of differentially expressed genes based on the association analysis of transcriptome and proteome. Table S11
