# Supplementary material for: USP45 acts as an oncogene to regulate the proliferation of esophageal cancer cells
Source: Genes Dis. 2023 Sep 30;11(5):101135. doi: 10.1016/j.gendis.2023.101135 (PMC11167240; doi:10.1016/j.gendis.2023.101135)
Supplement: Multimedia component 1 [file mmc1.doc]

**Construction of stable cell lines for USP45 overexpression and knockdown**

HEK293T, KYSE140 and KYSE410 cell lines were purchased from Institute of Cellular Resources, Shanghai Institutes for Biological Sciences, Chinese Academy of Sciences. HEK293T cells were cultured in DMEM medium, containing 10% fetal bovine serum (FBS; Gibco, 10099141), 1% penicillin and streptomycin. KYSE140 and KYSE410 cells were cultured in 1640 medium, containing 10% fetal bovine serum (FBS; Gibco, 10099141), 1% penicillin and streptomycin. The open reading frame sequence of USP45 was cloned into the lentiviral expression vector pReceiver-Lv120 (GeneCopoeia) to construct the USP45 overexpression plasmid with amplification forward primer: ATGCGGGTGAAAGATCCAACTAAAGC and reverse primer: TTATAATACTCTTTCATAGAAAAGAAG. Targeting the 5'-CCAACATGTAAGTCATGCTAT-3' sequence in USP45 mRNA by chemically synthesizing two sequences 5'- GATCCGCCAACATGTAAGTCATGCTATTCAAGAGATAGCATGACTTACATGTTGGTTTTTTTTGGAATT-3' and 5'- AATTCCAAAAAACCAACATGTAAGTCATGCTATCTTGAATAGCATGACTTACATGTTGGCGGATC-3'. Then the two sequences were mixed and annealed to form a double-stranded DNA structure. shRNA lentiviral expression vectors psi-LVRU6GP (GeneCopoeia) were cleaved using BamHI and EcoRI enzymes. The enzymatically cleaved psi-LVRU6GP empty vector was mixed with the double-stranded DNA structure to construct the USP45 shRNA plasmid using T4 DNA ligase. The constructed USP45 knockdown plasmids were verified by sequencing with forward sequencing primer 5'-TAATACGACTCACTATAGGG-3' and reverse sequencing primer 5'-CTGGAATAGCTCAGAGGC-3'. In addition, pReceiver-Lv120 and psi-LVRU6GP empty vectors were used as negative controls for USP45 overexpression and knockdown plasmids, respectively. USP45 overexpression and knockdown plasmids and their negative control plasmids were transfected into Lenti-Pac™ 293Ta packaging cells using the Lenti-Pac™ HIV Expression Packaging System (GeneCopoeia), respectively, and gently mixed and placed in a constant temperature incubator for 48 hours. The lentivirus particles in the supernatant were collected using the Lenti-Pac™ Lentivirus Concentration Reagent (GeneCopoeia) and the virus titer was detected using the Lenti-Pac™ HIV qRT-PCR Lentivirus Titer Assay Kit (GeneCopoeia). The collected lentivirus was used to infect esophageal cancer cells KYSE140 and KYSE410. The monoclonal cells were picked to be screened and expanded by adding puromycin in complete medium, and finally the construction of stable cell lines was confirmed by RT-qPCR and western blot assay.

**RT-qPCR and western blot**

The cells were collected, and RNA was extracted using Trizol reagent (Life Technologies, USA), and cDNA was synthesized using a reverse transcription kit (Biotool), and qPCR was performed by SYBR Green PCR Master Mix (Biotool) in CFX ConnectTM qPCR system (Bio-rad). The results were analyzed using the 2^-ΔΔct^ method. The primers used in the study included USP45 (Forward: GAATGTGCAAATATCTCCACGGT and Reverse: TGATCTGTCTCCCGTAAACTTCT), MYC (Forward: GTCAAGAGGCGAACACACAAC and Reverse: TTGGACGGACAGGATGTATGC), NCAPD3 (Forward: GTGCCAAGGTGGTAGATAAATCA and Reverse: AACATAGCGTATTCCCCACAAG), ATAD2 (Forward: GGAAAAACCTCGTCACCAGAG and Reverse: CGCCTGTTCATTCGTTTACAGTA), NCOA4 (Forward: GCTCAGCAGCTCTACTCGTTA and Reverse: GGCACACAGAGACTTGATTGG), STX3 (Forward: TCGGCAGACCTTCGGATTC and Reverse: TCCTCATCGGTTGTCTTTTTGC), GAPDH (Forward: CTGGGCTACACTGAGCACC and Reverse: AAGTGGTCGTTGAGGGCAATG). Proteins were extracted from cells using RIPA lysis buffer and using a BCA Protein Assay Kit (Beyotime Biotechnology Co.，Jiangsu，China) to measure concentration of proteins. Equal amounts of total protein (40μg) were loaded, run on 10% SDS-polyacrylamide gel and transferred to PVDF membranes (Millipore, Billerica, MA). The membranes were blocked with Tris-buffered saline containing 5% nonfat milk for 1 h, then probed with primary antibodies of target protein. USP45 antibody (AP13409c, dilution of 1:500) and GAPDH antibody (AM1020b, dilution of 1:2000) were purchased from Abcepta Biotech Company. c-myc antibody (AF6054, dilution of 1:500), NCAPD3 antibody (DF9413, dilution of 1:1000), ATAD2 antibody (DF14254, dilution of 1:500), NCOA4 antibody (DF4255, dilution of 1:500), STX3 antibody (DF12760, dilution of 1:500), KDELR2 antibody (DF4047, dilution of 1:500), Goat Anti-Rabbit IgG (H+L) HRP (S0001, dilution of 1:10,000) and Goat Anti-Mouse IgG (H+L) HRP (S0002, dilution of 1:10,000) were purchased from Affinity Biosciences Company.

**Cell colony formation assay and CCK-8 assay**

For the cell colony formation assay, the cells of each group in the logarithmic growth phase were digested with 0.25% trypsin and resuspended in complete medium separately. The cell suspensions of each group were diluted and inoculated at 100 cells/well into inoculated six-well plates. Gently rotate the culture dish to disperse the cells evenly. Place in a cell incubator at 37°C, 5% CO_2_ and saturated humidity. The growth of cell clones was observed at day 1, 5 and 8, respectively, using the time of cell apposition as day 1. Three fields of view were randomly selected through the microscope to record the cell clone size.

A CCK-8 assay was used to determine cell proliferation. For the CCK-8 assay (Cell counting kit-8, Dojindo Laboratories，China), cells (3×10^3^ cells/well) in the logarithmic growth phase were cultured in 96-well plates and incubated for 0 to 72 hours. After the treatment period, 10 µl CCK-8 was added and continued to culture 2 h. The value of OD450 was measured by automated microplated reader (BioTek, Winooski, VT, USA).

**Subcutaneous tumorigenesis experiment in nude mice**

KYSE410 stable cell lines of negative control (sh-NC) or USP45 knockdown (sh-USP45) were suspended in 100 µl of PBS solution and subcutaneously injected on the back of each group of mice (2×10^6^ cells/each mouse), respectively. The growth of subcutaneous tumors on the back of each group of mice was observed and recorded in real time (size, trait, texture, etc.), and tumor growth curves were plotted according to time and tumor volume. When the dorsal tumors grew to the appropriate size, the mice in each group were executed, and the appearance morphology, volume and weight of the subcutaneous tumors were observed and recorded. All animal experiments were approved by the Ethics Committee of Nanyang Institute of Technology.

**Transcriptome and proteomics analysis**

KYSE410 stable cell line of negative control (sh-NC) and USP45 knockdown (sh-USP45) in logarithmic growth phase were passaged to perform Transcriptome and proteomics analysis. The transcriptome analysis was performed by Beijing Biomarker Technologies, and the data analysis was performed on the online platform of Biomarker Cloud (www.biocloud.net). Proteomics analysis was performed by Shanghai Applied Protein Technology, and data analysis was performed on the online platform of APTBIOTECH BioCloud (https://bio-cloud.aptbiotech.com/).

**Statistical Analysis**

The experimental results are expressed in the form of mean ± standard deviation. Graphpad Prism 8.0 is used for statistical analysis. The statistical analysis between the two sets of data is performed by two-tailed Student’s t-test. P<0.05 was considered to indicate a statistically significant difference.
